# A Non-Enzymatic Sensor Based on Fc-CHIT/CNT@Cu Nanohybrids for Electrochemical Detection of Glucose

**DOI:** 10.3390/polym12102419

**Published:** 2020-10-20

**Authors:** Fang Wang, Sheng Hu, Fengna Shi, Kexin Huang, Jiarui Li

**Affiliations:** 1 Nanjing Forestry Univ, Coll Chem Engn, Nanjing 210037, Jiangsu, China; drwatson1995@163.com (S.H.); shi1355128616@163.com (F.S.); kexin0722@163.com (K.H.); lijiarui11251004@163.com (J.L.); 2 Nanjing Forestry Univ, Coinnovat Ctr Efficient Proc & Utilizat Forest Re, Nanjing 210037, Jiangsu, China

**Keywords:** carbon nanotube, chitosan, copper nanoparticles, ferrocene, electrochemical sensor, glucose, non-enzymatic

## Abstract

Herein, a composite structure, consisting of Cu nanoparticles (NPs) deposited onto carbon nanotubes and modified with ferrocene-branched chitosan, was prepared in order to develop a nonenzymatic electrochemical glucose biosensor ferrocene-chitosan/carbon nanotube@ Cu (Fc-CHIT/CNT@Cu). The elemental composition of the carbon nanohybrids, morphology and structure were characterized by various techniques. Electrochemical impedance spectroscopy (EIS) was used to study the interfacial properties of the electrodes. Cyclic voltammetry (CV) and chronoamperometry methods in alkaline solution were used to determine glucose biosensing properties. The synergy effect of Cu NPs and Fc on current responses of the developed electrode resulted in good glucose sensitivity, including broad linear detection between 0.2 mM and 22 mM, a low detection limit of 13.52 μM and sensitivity of 1.256 μA mM^−1^cm^−2^. Moreover, the modified electrode possessed long-term stability and good selectivity in the presence of ascorbic acid, dopamine and uric acid. The results indicated that this inexpensive electrode had potential application for non-enzymatic electrochemical glucose detection.

## 1. Introduction

Today, diabetes is a worldwide health problem. The literature states that there were 451 million (18–99 years) people around the world suffering from diabetes in 2017, and this number is estimated to be 693 million by 2045. Additionally, according to the estimation, almost half of diabetic patients (49.7%) do not get diagnosed [1]. Diabetes is a chronic disease that can cause some complications in severe cases, such as heart attack, stroke, kidney failure and nerve damage [2,3,4]. It is important to measure blood glucose levels during treatment of diabetes, especially in the early stages. In this case, new, more rapid, sensitive and selective glucose concentration detection methods are urgently needed. Using portable electronic devices to directly detect glucose is a convenient diagnostic method in the initial steps. A literature survey found that glucose sensors can be divided into two categories: enzyme-based sensors [5,6] and enzyme-free sensors [7,8]. Enzyme-based electrochemical biosensors are complex, expensive and affected by temperature and pH due to enzyme activity. Therefore, more concern has been brought to developing non-enzymatic electrochemical methods with high sensitivity, low detection limit, fast response speed, high reliability and good selectivity. In particular, various glucose sensors, based on nanomaterials’ electrocatalytic action, have made up for the deficiency of traditional enzyme-based sensors.

Metal nanoparticles, such as Au [9], Ni [10] and Co nanoparticles [11], are often used in construction of nonenzymatic sensors. Copper is an essential micronutrient for all living organisms, in addition to its good biocompatibility, low cost and environmentally friendly properties, which make copper nanoparticles attractive catalysts [12]. Carbon nanotubes are often used for metal nanoparticle support due to their relatively high chemical stability, large surface area, strong adsorption capacity and excellent electrical conductivity [13,14]. Work has been done using carbon nanotubes as a template to support metal nanoparticle catalysts [15]. In principle, metal nanoparticles are formed on the surface of carbon nanotubes, rather than on sidewall defect sites [16]. Therefore, the surface of carbon nanotubes needs to be functionalized to obtain uniformly distributed nanoparticles by covalent or noncovalent interactions [17,18].

Carrier–catalyst composites affect sensor performance in their manufacturing [19,20,21]. The performance of the carrier for immobilizing metal nanoparticles is one of the factors affecting the catalyst’s performance, because they affect dispersibility, conductivity and stability. These are the prerequisites for stable and effective sensor operation [19]. Natural polysaccharide chitosan (CHIT) is derived from deacetylation of chitin, and it has good film-forming ability, adhesion, excellent biocompatibility and high hydroxyl and amino content [22,23]. However, CHIT application in electrochemical sensors is limited due to its non-conductive properties. To improve its conductance, attempts to introduce metal elements were made. Ferrocene (Fc) and ferrocene-based derivatives have suitable electrochemical properties, such as stable redox state, reversibility and regeneration at low potential. Therefore, they are often used as good electronic conductors [24,25]. The purpose of adding Fc to the prepared composite material was to increase the electron transfer rate, thereby improving the electrochemical performance of the glucose sensor.

Herein, copper nanoparticles were decorated on acidified multiwalled carbon nanotubes (CNT@Cu) using a chemical reduction method. In pursuit of constructing a highly sensitive and wide linear range amperometric biosensor for glucose detection, a novel, enzyme-free glucose sensor was prepared using a glassy carbon electrode covered with ferrocene-branched chitosan/CNT@Cu nanohybrids. Because the materials had these advantages, the composite electrodes were fabricated to make use of their synergistic electrochemical effects.

## 2. Materials and Methods 

### 2.1. Materials

Chitosan was purchased from Beijing Solaribio Science & Technology Co. Ltd. (Beijing, China), with a degree of deacetylation > 90.0%. D-(+)-glucose anhydrous (≥98.0%), ferrocene formaldehyde, dopamine (≥98.0%), uric acid (≥98.0%) and ascorbic acid (≥98.0%) were purchased from Aladdin Chemical Reagent Inc. (Shanghai, China). MWCNTs (Multiwall carbon nanotubes) (OD < 8 nm, purity > 95%, ash < 1.5%) with a length of 10–30 μm were obtained from Xianfeng Nano Material Technology Co., Ltd. (Nanjing, China). Carboxylated MWCNTs were formed using an HNO_3_ solution. All other used chemicals were of analytical grade.

### 2.2. Apparatus

FT-IR spectra of all the samples were obtained and analyzed using a Fourier transform infrared instrument (FT-IR360, Nicolet, Wisconsin, WI, USA), using KBr pellets in the wavenumber range of 400–4000 cm^−1^. The UV spectrum of the samples was obtained by an ultraviolet spectrophotometer (SHIMADZU, Kyoto, Japan). A thermal analyzer (Q5000IR, Delaware, TA, USA) was used in the temperature range of 20–600 °C, heating rate of 10 °C/min and nitrogen flow rate of 10 mL/min to study thermal properties of pure chitosan, ferrocene and modified chitosan.

The internal feature of modified electrodes was characterized using a JEM-1400 transmission electron microscope (TEM, Tokyo, Japan). Before the test, a drop of diluted modified electrode material suspension was added onto a glassy carbon electrode (GCE), and a thin-film TEM test sample was obtained after drying at room temperature. Fc-CHIT/CNT@Cu nanohybrids were prepared for a thin film, and SEM images were obtained using a Hitachi S4800 scanning electron microscope.

Cyclic voltammetry (CV) (potential range was −0.8 to 0.8 V and scan rate was 10 mV/s), electrochemical impedance spectroscopy (EIS) (frequency range was 0.01–100,000 Hz), amperometric i-t curve (i-t) (potential window was 0.44 V) were all performed using a CHI 760E electrochemical workstation (Shanghai, China) with a conventional three-electrode system, which consisted of a glassy carbon electrode (the diameter was 3 mm) as the working electrode, a platinum wire as the counter electrode (CE) and a saturated calomel electrode (SCE) as the reference electrode. In this article, we used CV, EIS and i-t to test the electrochemical performance of different electrodes. In the i-t test, the initial electrolyte was a 20 mL NaOH solution (0.1 M), and the volume of the glucose solution was 10 μL for each addition.

### 2.3. Synthesis of Ferrocene-Branched Chitosan

Chitosan was modified with ferrocene according to the literature [26]. Briefly, 90 mg of CHIT was dissolved in 20 mL of acetic acid solution (1%). Fifteen milligrams of Fc was dissolved in 15 mL of methanol and then added into the CHIT solution and stirred for 1 h. After adding NaCNBH_3_, the reaction mixture was stirred for 24 h, and finally a 5% NaOH solution was added to the precipitate. The yellow product was collected and washed alternately with water and methanol. The solid product was freeze-dried to obtain Fc-CHIT.

### 2.4. Decoration of Cu NPs onto Carbon Nanotubes (CNT@Cu)

CNT@Cu nanocomposites were prepared using a chemical reduction method with a Cu salt and acidified multiwalled carbon nanotubes [27]. Firstly, 20 mL of CNT suspension (12.5 mg/mL) was added into a 20 mL CuSO_4_ (10 mmol/L) and 20 mL sodium citrate (40 mmol/L) solution, and magnetically stirred for 1 h. Then, a certain amount of 0.1 M NaBH_4_ solution was slowly added and stirred for another 1 h under ice-bath conditions. Finally, the obtained solution was washed, filtered and freeze-dried to obtain Cu-decorated CNT (CNT@Cu).

### 2.5. Preparation of the Modified Electrodes 

The modified electrode Fc-CHIT/CNT@Cu (Fc-CHIT/CNT, CHIT/CNT@Cu or CHIT/CNT) was prepared using a solution casting method. GCE was polished with 0.3 mm alumina powder and sonicated in ethanol and distilled water for 3 min each. Typically, 0.4 mg of CNT@Cu (or CNT) was introduced into 1 mL of Fc-CHIT (or CHIT) solution, and the mixture was sonicated for 1 h. After that, 5 μL suspended droplets were added to the surface of GCE, and the electrode was dried at room temperature. Thus, a nonenzymatic electrochemical biosensor, based on CHIT/CNT nanohybrids, was obtained. 

## 3. Results

### 3.1. Characterization of Composites

**Figure 1:**
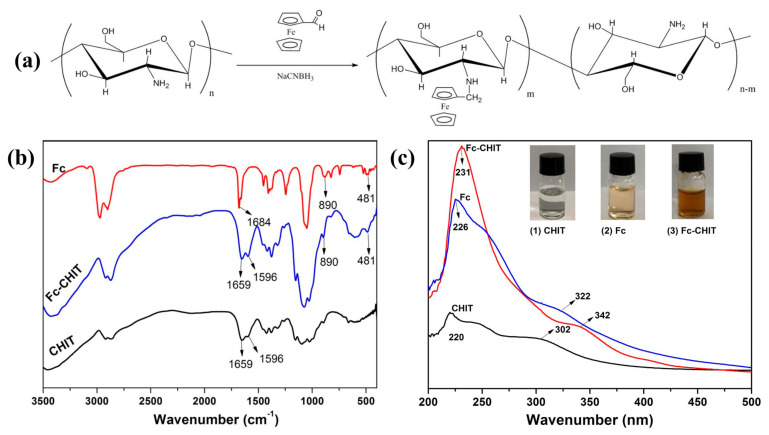
(**a**) Reaction scheme of CHIT and Fc; (**b**) FT-IR and (**c**) UV spectra of CHIT, Fc and Fc-CHIT.

The chitosan modification reaction and resulting product analysis are shown in Figure 1. From the FT-IR spectra in Figure 1a, the chitosan spectrum showed characteristic peaks at 1659 cm^−1^ and 1596 cm^−1^, which were related to the amide I and II bands of CHIT. The aldehyde group in Fc-CHO reacted with the amino group in chitosan, and a covalent bond was formed by Schiff-based reaction (i.e., –C–N–) [26]. Therefore, the Fc-CHO characteristic peak at 1684 cm^−1^ disappeared in the Fc-CHIT spectrum. At the same time, the Fc-CHIT spectrum showed a new absorption band at 481 cm^−1^ due to the M-ring extension and ring inclination of the ferrocene group. Furthermore, another absorption band at 890 cm^−1^ also indicated that the ferrocene group was present in Fc-CHIT. The absorption peaks between 2860 cm^−1^ and 2970 cm^−1^ in all three samples were due to the existence of –CH_3_ and –CH_2_, while the peak around 1070 cm^−1^ was the C–O absorption band. Furthermore, the UV spectra of CHIT, Fc and Fc-CHIT also indicated successful synthesis of Fc-CHIT, shown in Figure 1c [28]. From the spectra, CHIT and Fc-CHO had characteristic absorption peaks at 220 and 302 nm as well as 231 and 342 nm, respectively. However, Fc-CHIT showed absorption bands at 226 nm and 322 nm, which was caused by an Fc group grafted to chitosan. 

**Figure 2:**
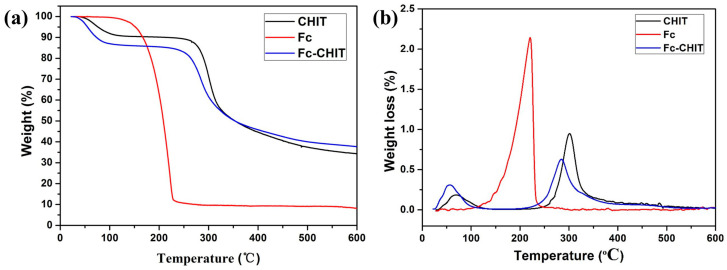
Thermogravimetric analysis before and after Fc-modified chitosan; (**a**) TGA, (**b**) DTG.

Thermogravimetric analysis of Fc-CHIT is shown in Figure 2, which also shows TGA and DTA data of Fc-CHO and chitosan. In the range of 30 °C to 100 °C, due to evaporation of water, the pristine CHIT showed small weight loss. However, there was rapid weight loss when it decomposed at 302 °C. On the other hand, Fc showed large decomposition around 220 °C. The TG curve of Fc-CHIT was similar to that of CHIT, but it lost more weight due to decomposition of Fc before 300 °C. The difference between TG curves indicated that the proportion of Fc in Fc-CHIT was about 5.1 wt%.

**Figure 3:**
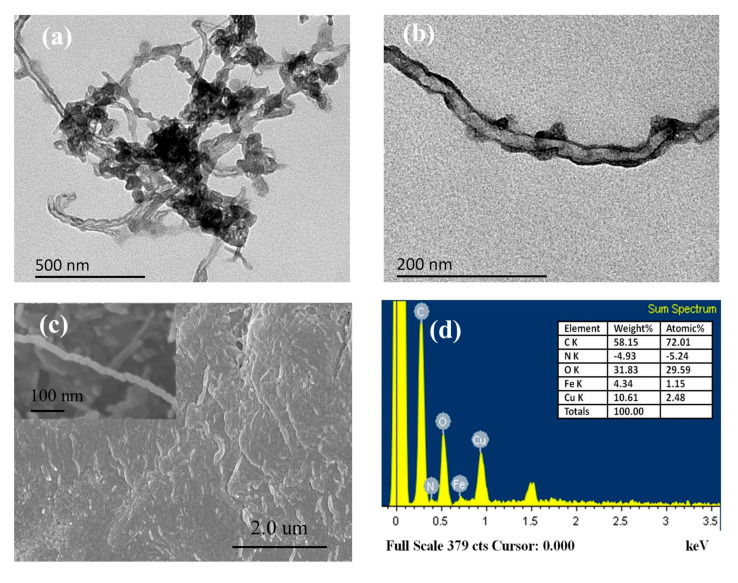
(**a**) TEM images of Fc-CHIT/CNT@Cu (**b**) under high magnification; (**c**) SEM images of Fc-CHIT/CNT@Cu and (**d**) energy dispersive X-ray spectrum.

Microstructural morphology of Fc-CHIT/CNT@Cu composites are characterized and shown in Figure 3 using TEM and SEM. The observed agglomeration in the TEM image in Figure 3a might have resulted from the electron donor–acceptor interaction in the Fc-CHIT/CNT@Cu composite structure. In this work, Cu NPs were specifically dispersed into the CNT network in order to fabricate uniform Fc-CHIT/CNT@Cu composites. In Figure 3c, spherical spots appeared in the SEM image, which was the manifestation of successful Cu NP formation on the surface of carbon nanotube composites. From energy-dispersive spectra (EDS) of the composites (Figure 3d), it could be clearly seen that C, N, O, Cu and Fe were the main components. Therefore, EDS results also showed that Cu NPs were successfully deposited, and Fc was incorporated into the composites. SEM of pristine CNT and Fc-CHIT/CNT are shown in Figure S1, which could not be seen in the presence of Cu.

### 3.2. Electrochemical Measurement

#### 3.2.1. Electrochemical Characterization of Modified Electrode

**Figure 4:**
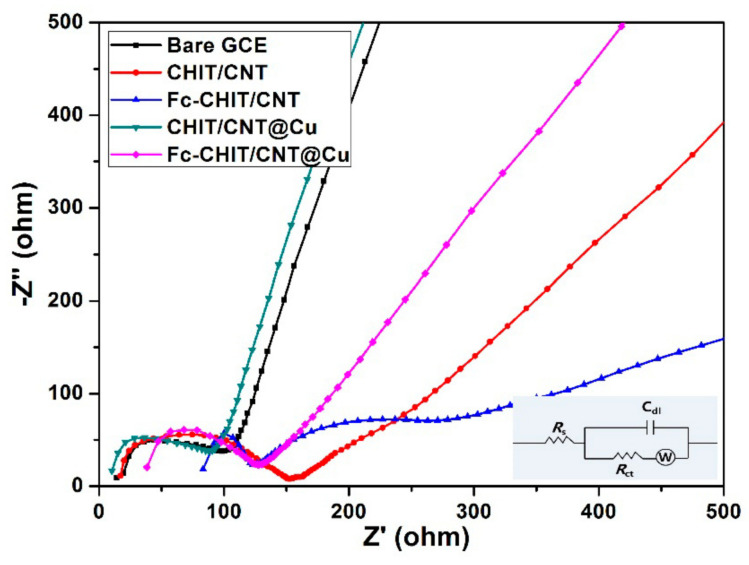
Nyquist plots for bare GCE, CHIT/CNT, CHIT/CNT@Cu, Fc-CHIT/CNT and Fc-CHIT/CNT@Cu (inset is an equivalent circuit model). Electrolyte: 5.0 mM Fe(CN)_6_^3−^/Fe(CN)_6_^4−^ containing 0.1 M KCl.

Electrode surface impedance changes during the modified process could be determined using electrochemical impedance spectroscopy (EIS). All of their Nyquist plots, except Fc-CHIT/CNT, consisted of an inclined line at the low-frequency region and a semicircular part at high frequency, corresponding to the diffusion-limited process and the electron transfer limiting process, respectively. The results are shown in Figure 4. EIS plot for Fc-CHIT/CNT alone showed two semicircles that might correspond to charge transfer resistance levels at the edges and at the center [29]. The high frequency semicircle affected electron transfer dynamics in the interface of electrode/electrolyte, and its diameter corresponded to charge transfer resistance (Rct). It could be clearly observed that the CHIT/CNT showed larger Rct than bare GCE, which indicated that CHIT/CNT did not improve conductivity on the surface of GCE. On the other hand, it is worth noting that the Rct value of Fc-CHIT/CNT and CHIT/CNT@Cu was lower than that of CHIT/CNT, which indicated that the presence of Fc and Cu NPs could improve conductivity of the composites. Further, the Rct value of Fc-CHIT/CNT@Cu was the smallest, which showed that Fc and Cu NPs had a synergistic effect in improving conductivity. Through software analysis, Rct values of CHIT/CNT, Fc-CHIT/CNT, CHIT/CNT@Cu and Fc-CHIT/CNT@Cu were 138.60, 56.56, 0.38 and 0.14 Ω, respectively, which confirmed the above conclusions. This also indicated that conductive Fc and Cu NPs were successfully immobilized onto chitosan/CNT nanocomposites. 

**Figure 5:**
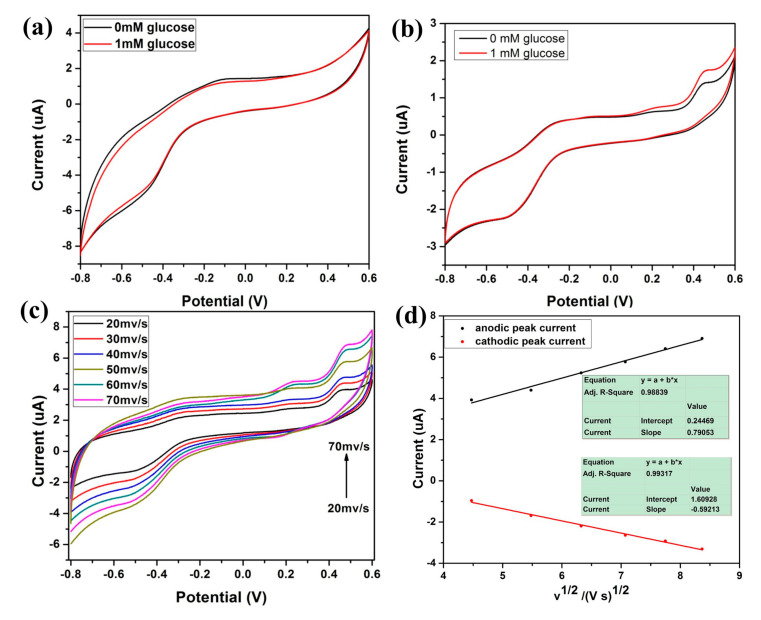
CVs of (**a**) CHIT/CNT and (**b**) Fc-CHIT/CNT@Cu electrodes in 0.1M NaOH electrolyte without glucose and with 1 mM glucose. (**c**) CVs of the Fc-CHIT/CNT@Cu electrode in 0.1M NaOH electrolyte at scan rates of 20, 30, 40, 50, 60 and 70 mV/s. (**d**) Plots of anodic and cathodic peak currents versus scan rates (v^1/2^).

CV was performed to study Fc-CHIT/CNT@Cu electrode electro-oxidation performance towards glucose in 0.1 M NaOH electrolyte. The potential range was −0.8 V to 0.6 V at a scan rate of 10 mV s^−1^. As shown in Figure 5a, the CHIT/CNT electrode had no obvious redox peaks with 1 mM glucose or without glucose, confirming that CHIT/CNT could not detect glucose electrochemically. Figure 5b shows, however, that after CHIT/CNT were modified with Fc and Cu NPs, the carbon nanohybrid electrode had three anodic peaks at around −0.3, 0.4 and 0.2 V, which corresponded to Cu/Cu (II), Cu (II)/Cu (III) and Fe (II)/Fe (III) redox couples. After adding 1 mM of glucose into a 0.1 M NaOH solution, the peak anodic current increased, indicating that the Fc-CHIT/CNT@Cu electrode had a response towards glucose detection. CVs and i-t test of CHIT/CNT@Cu, Fc-CHIT/CNT and Fc-CHIT/CNT@Cu in Figures S2 and S3 indicated that Cu and Fc had a synergistic catalysis effect to glucose. Some studies have shown that under alkaline conditions, electro-oxidation of glucose on the surface of Cu is usually accompanied by chemical valence state transition of Cu [4,30,31,32,33]. Firstly, Cu is oxidized to Cu (II) (Equations (1)–(2)). Subsequently, Cu (II) is oxidized to Cu (III), such as Cu_2_O_3_ and CuO^2−^ (Equations (3)–(4)), and Fe (II) is oxidized to Fe (III) (Equation (5)). Finally, after glucose is added to the alkaline electrolyte solution, it is oxidized to gluconolactone (Equation (6)).

Cu + 2OH^−^ → Cu (OH)_2_ + 2e^−^ (1)

Cu (OH)_2_ ↔ CuO + H_2_O (2)

2CuO + 2OH^−^ ↔ Cu_2_O_3_ + H_2_O + e^−^ (3)

2Cu (OH)_2_ + 2OH^−^ ↔ CuO_2_^−^ + H_2_O + e^−^ (4)

Fe(II) ↔ Fe(III)(5)

(6) $$\mathrm{glucose}\;\overset{\mathrm{Cu}(\mathrm{III})/\mathrm{Fe}(\mathrm{III})}{↔}{\;\mathrm{gluconolactone}\;+\;e}^{-}$$

CVs at the Fc-CHIT/CNT@Cu electrode in 0.1 M NaOH solution at different scan rates of 20 to 70 mv/s are shown in Figure 5c. It displayed clearly that the peak shifted to a higher potential value as the scan rate increased. Further, the relationship between scanning rate and electrochemical behavior was also evaluated and is shown in Figure 5d. As the square root of the scan rate increased, the peak current at 0.44 V and −0.5 V increased linearly. The results indicated that oxidation of glucose on the electrode surface was a diffusion-controlled reaction [34,35]. Figure S4 indicate the electrolyte pH and constituents will cause current values change with the same glucose concentration detection.

#### 3.2.2. Electrochemical Activity of Fc-CHIT/CNT@Cu towards Glucose Detection

**Figure 6:**
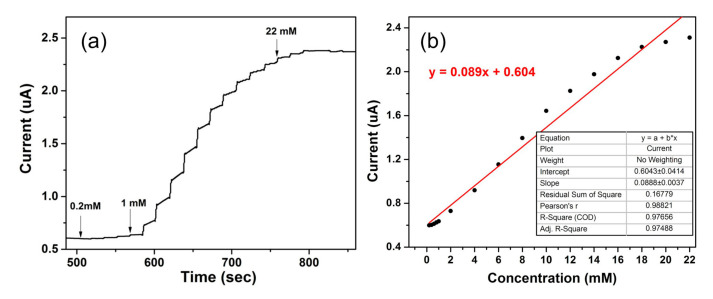
(**a**) Amperometric i-t curve response of serial concentrations of glucose obtained at the Fc-CHIT/CNT@Cu electrode. The difference in the concentration of glucose was marked with arrow lines. (**b**) Plot for linear variation of current vs. glucose concentration of range of Fc-CHIT/CNT@Cu electrode (in glucose concentration range of 0.2–22 mM). Conditions: supporting electrolyte, 0.1 M NaOH; operating potential, 0.44 V.

Analytical characterization of Fc-CHIT/CNT@Cu/GCE, based on chronoamperometric measurements, was performed, and the applied potential was equal to 0.44 V, based on the CV curve. The results in Figure 6 showed that after adding a glucose solution, the oxidation current could reach a steady state within 2 s. There was a linear relationship between the oxidation current value and glucose concentration in the range of 0.2 mM to 22 mM. The calibration curve for glucose concentration was shown as Ip (μA) = 0.120 [glucose] (mM) + 0.810 (μA). Sensitivity of the proposed sensor was calculated as 1.256 uA mM^−1^ cm^−2^. Good sensitivity could be attributed to the synergy between ferrocene and Cu NPs. When the signal-to-noise ratio was 3 (S/N=3), the limit of detection (LOD) was determined from the linearity range of the calibration curve based on the following equation:

(7) $$LOD=\frac{3\sigma }{s}$$

where *σ* is the standard deviation of blank solution (11 readings), and *s* is the slope of the chronoamperometric calibration curve. LOD of the proposed sensor was 13.52 μM. Compared with other electrochemical methods described in literature (Table 1), the detection limit of our proposed biosensor for glucose detection remained at a similar level with high sensitivity.

#### 3.2.3. Selectivity and Stability of the Biosensor

The amperometric response of the Fc-CHIT/CNT@Cu sensor with the addition of typical biological interferences, such as ascorbic acid (AA), dopamine (DA) and uric acid (UA), to the glucose solution was determined in order to evaluate the biosensor’s selectivity. Compared with normal physiological levels of glucose (3–8 mM), the interfering species’ levels were much lower, such as AA (about 0.1 mM), DA (about 0.02 mM), UA (about 0.02 mM) and so on [47]. 

**Figure 7:**
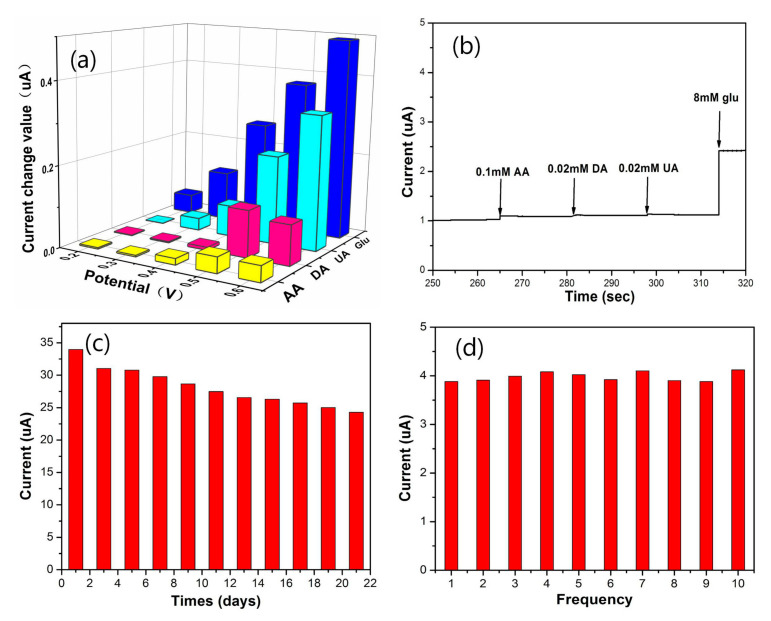
Current values of the Fc-CHIT/CNT@Cu electrode with the addition of ascorbic acid (AA), dopamine (DA), uric acid (UA) and glucose (0.1 M NaOH) (**a**) under different applied potentials; (**b**) under 0.44 V applied potential; (**c**) study of sensor stability over 7 days with addition of 4 mM glucose; (**d**) repeatability was measured by 10 successive measurements of the same sensor at 0.44 V potential, with addition of 4 mM glucose.

The current value changes were tested at different potentials (Figure 7a) with the addition of AA, DA, UA or 1 mM glucose. It could be seen that there was almost no response current of AA and DA below 0.4 V, and all current values increased to a higher potential. However, the three interferences caused slight current changes when compared to the addition of glucose. As shown in Figure 7b, under 0.44 V working potential, the addition of 0.1 mM AA caused a slight current response, and there were no obvious responses to 0.02 mM DA and UA. However, after adding 8 mM glucose, the sensor had an obvious response. Figure S5 is the selectivity of the elctrodes with different electrolyte pH and constituents. These results indicated that Fc-CHIT/CNT@Cu was beneficial to the conductivity of the enzyme-free glucose sensor with good selectivity, and it could reduce the interference of electroactive substances, which is more favorable to practical applications.

The long-term stability of glucose detection of the modified electrode was then assessed. The Fc-CHIT/CNT@Cu-modified electrode was stored at 4 ^o^C during this study. The current response to 4 mM glucose was checked every day using amperometry, after the baseline was tested in a 0.1 M NaOH electrolyte solution. As shown in Figure 7c, the current retained about 94% of the initial value after seven days, which indicated that Fc-CHIT/CNT@Cu could be regarded as a stable glucose sensor. Reliable reproducibility was also an important part of evaluating the sensor’s actual performance. As seen in Figure 7d, the RSD was only 3.3%, which indicated that the sensor showed good reproducibility.

## 4. Conclusions

In the current study, ferrocene was covalently linked with chitosan via the Schiff-base reaction. In the next step, using a chemical reduction method, we obtained CNT@Cu nanohybrids. The most prominent achievement of this work was the construction of a ferrocene-branched chitosan/CNT@Cu/GCE nanohybrid biosensor for enzyme-free glucose determination. The structure and morphology of the composite electrode were studied. The electrode showed excellent glucose detection performance (wide linear range, low detection limit, high sensitivity), which was due to the large specific surface area of the carbon nanotube, good conductivity of Cu NPs and a certain synergistic effect of Fc with unique redox behavior. In addition, the sensor had good selectivity and long-term stability. Therefore, the Fc-CHIT/CNT@Cu electrode has promising potential for glucose detection.

## Figures and Tables

**Table 1:** Comparison of glucose detection using various sensing electrodes.

Modified Electrode	LOD (μM)	Linear Range (mM)	Sensitivity (μAmM^−1^cm^−2^)	Electrolyte pH	Ref.
Cu@CHIT–CNT	5 × 10^−2^	0.5 × 10^−3^~1	Not mentioned	12.7	[36]
AuNPs-MWCNTs-CHIT cryogel	0.5	1 × 10^−3^~1	Not mentioned	12.7	[37]
Cu/NiNPs/CMWCNTs-ITO	0.67	1 × 10^−3^~1	6.782	13	[31]
rGO-Fc/GA-GOx/GCE	2 × 10^−2^	2~10 × 10^−3^	Not mentioned	7.4	[6]
CuNPs/NGO	0.44	0.001~1.803	2500	13	[38]
Cu/CuO/ZnO	18	0.1~1	408	13	[39]
AuCu/CNTs	4	0.08~9.260	22	13	[40]
Cu NPs/SWCNTs	0.3	0.5~500 × 10^−3^	0.256	12.3	[41]
Cu/Co NWs	5 × 10^−2^	0.3~2.6 × 10^−3^	0.097	13.7	[42]
Cu nanoporous	40	0.01~0.5	220	13.3	[43]
S-rGO/CuS	0.032	up to 20.17	429.4	13	[44]
CuO-6	0.307	up to 5.664	992.073	12.7	[45]
CuO-CS/GCE	11	0.05~1	503.129	13	[46]
Fc-CHIT/CNT@Cu/GCE	13.52	0.2~22	1.256	13	This work

## Supplementary Materials

The following are available online at https://www.mdpi.com/2073-4360/12/10/2419/s1, Figure S1: SEM of (a) pristine CNT and (b) Fc-CHIT/CNT, inset images are the EDS and element content, Figure S2: CVs of Fc-CHIT/CNT, CHIT/CNT@Cu and Fc-CHIT/CNT@Cu electrodes in 0.1 M NaOH electrolyte (a) without glucose and (b) with 1 mM glucose, Figure S3: The relationship between glucose concentration and current value of CHIT/CNT@Cu, Fc-CHIT/CNT and Fc-CHIT/CNT@Cu modified GCE electrodes, Figure S4. (a) Chronoamperograms obtained with different NaOH concentrations and 0.1 M KOH; (b) Current values of NaOH concentrations or 0.1 M KOH with 4 mM glucose, Figure S5: The current response of the Fc-CHIT/CNT@Cu electrode to the addition of AA, DA, UA, glucose with different electrolyte pH and constituents.
