# Supplementary material for: A Non-Enzymatic Sensor Based on Fc-CHIT/CNT@Cu Nanohybrids for Electrochemical Detection of Glucose
Source: Polymers (Basel). 2020 Oct 20;12(10):2419. doi: 10.3390/polym12102419 (PMC7589752; doi:10.3390/polym12102419)
Supplement: Supplementary file 1 [file polymers-12-02419-s001.pdf]

polymers

Article

A Non-Enzymatic Sensor Based on Fc-CHIT/CNT@Cu Nanohybrids for Electrochemical Detection of Glucose

Fang Wang [1,2,*], Sheng Hu [1], Fengna Shi [1], Kexin Huang [1] and Jiarui Li [1]

[1] Nanjing Forestry Univ, Coll Chem Engn, Nanjing 210037, Jiangsu, China; drwatson1995@163.com (S.H.); shi1355128616@163.com (F.S.); kexin0722@163.com (K.H.); lijiarui11251004@163.com (J.L.)
[2] Nanjing Forestry Univ, Coinnovat Ctr Efficient Proc & Utilizat Forest Re, Nanjing 210037, Jiangsu, China.
* Correspondence: wangfang@njfu.edu.cn

Figure S1 is the SEM images and Energy dispersive spectra (EDS) of pristine CNT. From the results, it could be not seen the presence of Cu element in the two pictures.

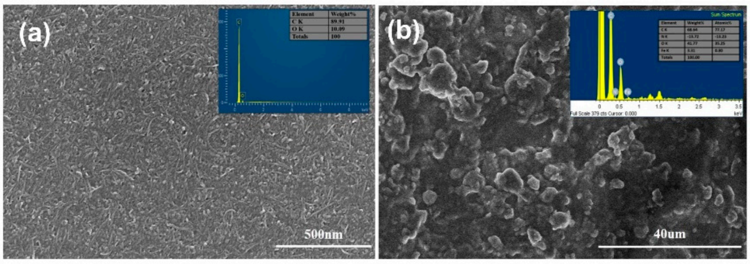

Figure 1. SEM of (a) pristine CNT and (b) Fc-CHIT/CNT, inset images are the EDS and element content.

Figure S2 show the CVs using the Fc-CHIT/CNT and the CHIT/CNT@Cu. The results indicate the synergetic catalysis of Cu and Fc in Fc-CHIT/CNT@Cu electrode.

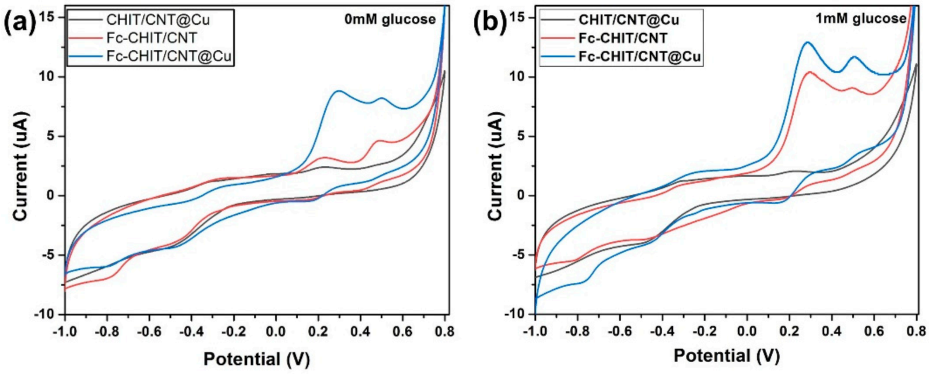

Figure 2. CVs of Fc-CHIT/CNT, CHIT/CNT@Cu and Fc-CHIT/CNT@Cu electrodes in 0.1 M NaOH electrolyte (**a**) without glucose and (**b**) with 1 mM glucose.

We have also compared CHIT/CNT@Cu, Fc-CHIT/CNT and Fc-CHIT/ CNT@Cu modified GCE elctrodes for the detection of glucose between 2-10 mM, the results are shown in Figure S3. The results indicate Fc-CHIT/CNT@Cu has higher current value than the other two electrodes under the same glucose concentration, which is due to the synergetic catalysis of Cu and Fc. Therefore, Fc-CHIT/CNT@Cu shows good sensitivity towards glucose detection.

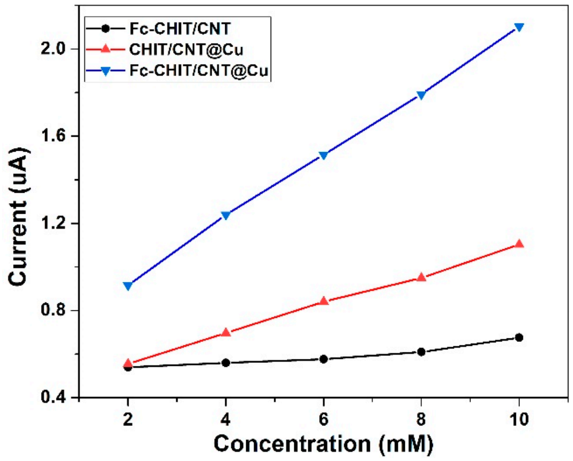

Figure 3. The relationship between glucose concentration and current value of CHIT/CNT@Cu, Fc-CHIT/CNT and Fc-CHIT/CNT@Cu modified GCE electrodes.

Figure S4 show the effect of other pH media towards 4 mM glucose detection. The results indicate the higher NaOH concentration, the higher current towards glucose detection. Fig. S4b indicate with the same electrolyte concentration (0.1 M), KOH can cause higher current than NaOH towards glucose detection, which is due to higher alkaline of KOH solution.

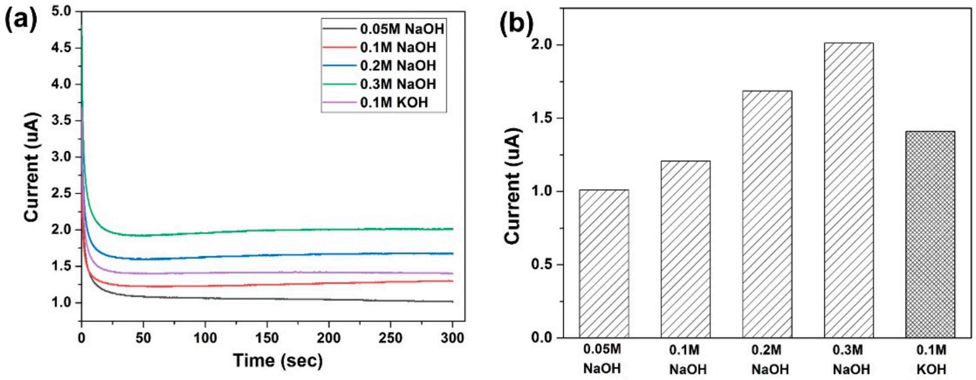

Figure 4. (**a**) Chronoamperograms obtained with different NaOH concentrations and 0.1 M KOH; (**b**) Current values of NaOH concentrations or 0.1 M KOH with 4 mM glucose.

We have also done the selectivity experiments of Fc-CHIT/ CNT@Cu modified GCE elctrodes with different electrolyte pH and constituents, the results are shown in Figure S5. Compared with control electrolyte solutions, addition 4 mM glucose would cause significantly increased current values. However, the addition of AA, DA and UA would cause a little current response . The results indicate the modified electrode has good selectivity for glucose detection.

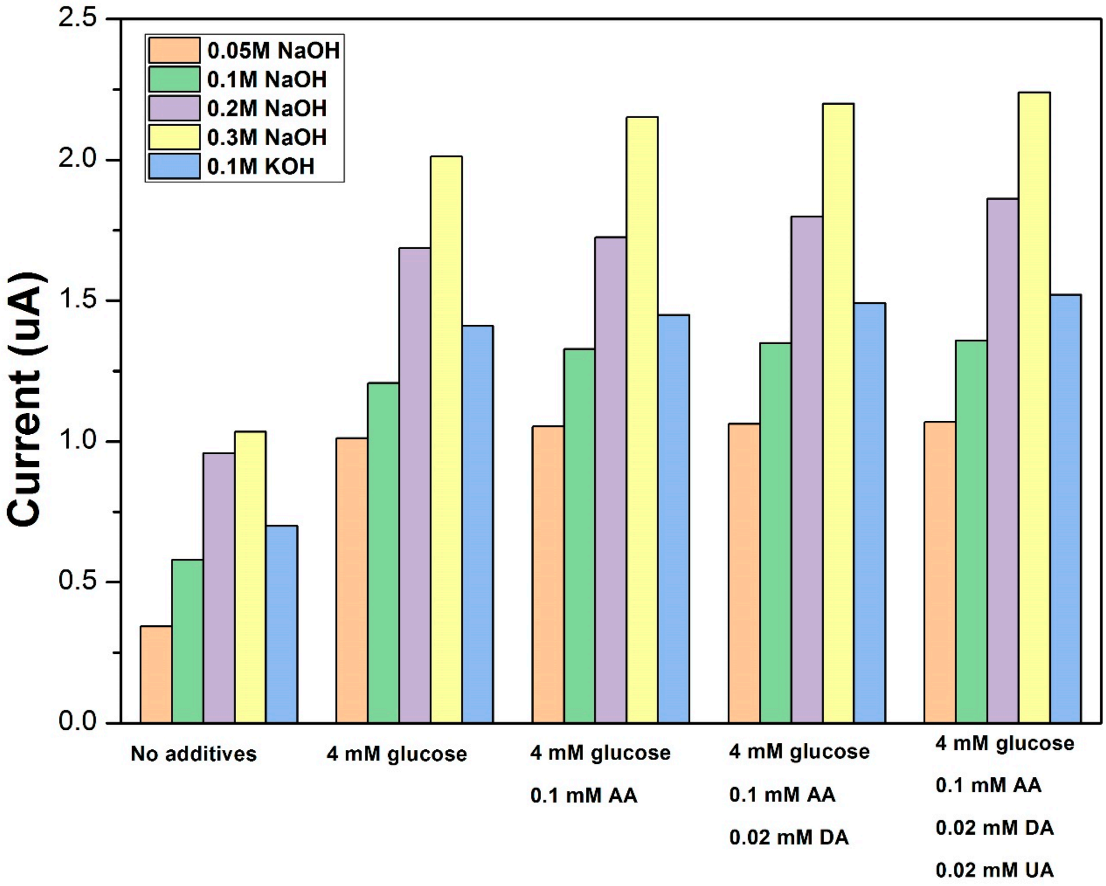

Figure 5. The current response of the Fc-CHIT/CNT@Cu electrode to the addition of AA, DA, UA, glucose with different electrolyte pH and constituents.
